# Elucidating the causal effects of plasma metabolites on breast cancer from multiple perspectives

**DOI:** 10.1097/JS9.0000000000003544

**Published:** 2025-09-29

**Authors:** Kun Fang, Yuhang Zhou, Shuo Wu, Dapeng Li, Xiaoli Cui, Shulan Sun, Xiang Li, Chang Liu

**Affiliations:** a Central Laboratory, Cancer Hospital of China Medical University; Cancer Hospital of Dalian University of Technology; Liaoning Cancer Hospital & Institute, Shenyang, Liaoning Province, The People’s Republic of China; b Department of Pharmacology, Cancer Hospital of China Medical University; Cancer Hospital of Dalian University of Technology; Liaoning Cancer Hospital & Institute, Shenyang, Liaoning Province, The People’s Republic of China; c Department of Breast Medicine, Cancer Hospital of China Medical University; Cancer Hospital of Dalian University of Technology; Liaoning Cancer Hospital & Institute, Shenyang, Liaoning Province, The People’s Republic of China; d Liaoning Promotion Center for the Transformation of Scientific and Technical Achievements, Liaoning Scientific and Technical Innovation Service Center, Shenyang, Liaoning Province, The People’s Republic of China; e Department of Gynaecology, Cancer Hospital of China Medical University; Cancer Hospital of Dalian University of Technology; Liaoning Cancer Hospital & Institute, Shenyang, Liaoning Province, The People’s Republic of China; f Department of Breast Surgery, Cancer Hospital of China Medical University; Cancer Hospital of Dalian University of Technology; Liaoning Cancer Hospital & Institute, Shenyang, Liaoning Province, The People’s Republic of China.

**Keywords:** breast cancer, bulk RNA, causal inference, Mendelian randomization, plasma metabolites, single-cell RNA

## Abstract

**Background:**

Early studies found common metabolic reprogramming in breast cancer. However, the causal effects of plasma metabolites on breast cancer remain unclear.

**Methods:**

Mendelian randomization (MR) and linkage disequilibrium score (LDSC) regression were performed based on previously published genome-wide association study (GWAS) summary statistics. The robustness of the causal inference was validated by sensitivity analyses. Gene mapping was performed by combining significant single-nucleotide polymorphisms (SNPs) and expression quantitative trait loci (eQTL) data to obtain the related gene set. Immune-related genes were identified by bulk and single-cell RNA analyses. Clinical samples were used for validation.

**Results:**

After correction and sensitivity analysis, seven significant plasma metabolites were identified. LDSC did not detect significant genetic correlations. Mapping SNPs to genes showed that two genes (SPP1 and ADM2) were significantly upregulated in breast cancer. However, only SPP1 passed external cohort validation. Similar results were obtained in the single-cell RNA analysis. In addition, SPP1 was found to be more highly expressed in monocytes. In clinical samples of breast cancer, we verified the high expression of SPP1 in monocytes–macrophages. At the cellular level, the high expression of SPP1 in monocytes–macrophages can promote malignant phenotypes such as proliferation and migration of breast cancer cells, and its expression level is regulated by myristic acid in significantly differential plasma metabolites.

**Conclusion:**

The findings provided valuable insight for the development of personalized treatment strategies for breast cancer and indicate that SPP1 may be a therapeutic target.

**HIGHLIGHTS**

- Multiple genetic techniques identified seven plasma metabolites with reliable causal effects on breast cancer.
- A multilayered body of evidence, including bulk RNA, scRNA sequencing, and clinical samples validation, identified SPP1 as a preliminary candidate target.
- The potential drugs and adverse reactions targeting SPP1 were predicted.

## Introduction

Breast cancer is the most common cancer in women worldwide, and different subtypes require different treatment approaches^[1]^. According to the latest global cancer statistics, in 2022, there were an estimated 2 308 897 new cases of breast cancer in women worldwide (accounting for 11.6% of all cancer cases) and 665 684 cancer-related deaths (accounting for 6.9% of all cancer deaths)^[2]^. Although some of the mechanisms underlying breast cancer have been elucidated, they are not yet fully understood. Thus, further exploration of targets and the pathogenesis of breast cancer will provide valuable insight for the development of new prevention strategies and treatment approaches.

Many substrates and products generated by enzyme-catalyzed reactions are present in plasma. These metabolites, which are present in the systemic circulation, may actively contribute to the initiation and progression of breast cancer. Recent advances in metabolomics have led to studies focusing on circulating metabolites to explore their relationship with breast cancer risk. Lipids differ significantly between the plasma of breast cancer patients and healthy individuals^[3]^. Moreover, reports indicate that plasma metabolites can serve as biomarkers for breast cancer and exhibit good diagnostic performance^[4,5]^. The 27-hydroxycholesterol, the most abundant cholesterol oxidative metabolite in plasma, acts as a selective estrogen receptor modulator that promotes the initiation and progression of breast cancer^[6]^. Overall, these studies highlight the important role of plasma metabolites in the pathogenesis of breast cancer. However, studies have been limited by the number of participants, insufficient metabolites analyzed, and the influence of confounding factors, and effective causal relationships remain to be established.

In recent years, based on the large amount of data accumulated from early genome-wide association studies (GWAS), Mendelian randomization (MR) analysis and linkage disequilibrium score (LDSC) regression were proposed to estimate causal effect and genetic correlations between traits^[7–9]^. MR has gained widespread attention in the medical field by inferring causal relationships between traits using single-nucleotide polymorphisms (SNPs) as instrumental variables. LDSC regression can determine the genetic correlations between traits from GWAS summary statistics and assess whether the results of MR are confounded by common genetic components.

We systematically assessed the causal effects and genetic correlations between plasma metabolites and breast cancer risk within the framework of MR and LDSC regression. We also mapped the genetic instruments involved in MR to genes to elucidate the potential molecular mechanisms by which significant plasma metabolites regulate breast cancer risk, and to identify new candidate targets for breast cancer.

## Methods

This study adheres to the Transparency In The Reporting of Artificial Intelligence (TITAN) guideline (Supplementary Digital Content Table S1, available at: http://links.lww.com/JS9/F233)^[10]^.

### Study design of MR

Under the MR framework, we examined the causal effects of plasma metabolites on breast cancer. MR analysis relies on three assumptions^[11]^: (1) genetic instruments are strongly associated with plasma metabolites, (2) genetic instruments are independent of other unmeasured phenotypes, and (3) genetic instruments are associated with breast cancer risk only through plasma metabolites. The validity of the causal effect can only be ensured if the above assumptions are satisfied. This study followed the Strengthening the Reporting of Observational Studies in Epidemiology-MR (STROBE-MR) guidelines^[12]^ and adhered to the key principles of the strengthening the STROBE guidelines (Supplementary Digital Content Table S2, available at: http://links.lww.com/JS9/F233). Given that the study used publicly available GWAS summary data, no additional ethical review was required.

### Data sources

GWAS summary statistics were obtained for 1091 plasma metabolites and 309 metabolite ratios from the Canadian longitudinal study on aging (CLSA) cohort^[13]^. The study involved 8299 elderly participants (62.4 ± 9.9 years old, all of European descent), ensuring that subsequent analyses were not significantly affected by a small sample size. Plasma metabolites refer to the metabolites measured in plasma samples, the majority of which are substrates or products generated by enzyme-catalyzed reactions. Metabolite ratios are the ratio of metabolite levels in pairs of metabolites that share enzymes or transport proteins recorded in the human metabolome database (HMDB). Some metabolites are derived from characterized molecules; 220 metabolites starting with “X” represent unidentified metabolites. GWAS summary statistics for plasma metabolites can be obtained from the GWAS Catalog (https://gwas.mrcieu.ac.uk/, accession numbers: GCST90199621 to GCST90201020).

GWAS summary statistics for breast cancer were obtained from the Breast Cancer Association Consortium (BCAC), which conducted an extensive GWAS analysis on 228 951 individuals of European ancestry, including 122 977 cases and 105 974 healthy controls^[14]^. Breast cancer data can be downloaded from the IEU Open GWAS project (https://gwas.mrcieu.ac.uk/datasets, accession number: ieu-a-1126).

### Selection of genetic instruments

Before the bidirectional MR analysis, the following criteria were established to select and harmonize genetic instruments: (1) based on previous studies^[15,16]^, a genome-wide significance threshold of *P* < 1e-05 was established to extract genetic instruments associated with plasma metabolites, (2) clustering was performed using *r*^2^ < 0.001 and a window size of 10 000 kb to exclude the effects of linkage disequilibrium, (3) only genetic instruments with effect allele frequencies (EAF) > 0.01 were included, (4) palindromic SNPs were removed, (5) genetic instruments with an *F*-statistic < 10 were excluded to avoid weak instrument bias, and (6) LDlink (https://ldlink.nih.gov/) was used to investigate SNPs within *r*^2^ = 0.1 and a 50 kb window surrounding genetic instruments to exclude pleiotropic genetic instruments associated with smoking and alcohol consumption, type 2 diabetes, education status, and body mass index.

### MR statistical analysis

Characterized by the use of regression without an intercept and employing the inverse of the outcome variance as weights, this method integrated the Wald estimates of each SNP, which helped reduce the influence of confounding factors and enhanced the accuracy of the results^[17]^. Therefore, inverse variance weighted (IVW) was used as the primary analytical method. The analysis was supplemented with MR-Egger and Weighted Median to validate the risk trends observed with IVW. The robustness of the results was increased by performing extensive sensitivity analyses. Cochran’s Q test can assess the heterogeneity of genetic instruments^[18]^. When heterogeneity is present, random-effects IVW is used for statistical analysis, whereas fixed-effects IVW is applied in the absence of heterogeneity. Scatter plots can help visually identify the presence of outliers, and leave-one-out analysis can further validate these results^[19]^. Horizontal pleiotropy is critical to the results of MR. Therefore, the Egger intercept test and pleiotropy residual sum and outlier (PRESSO) global test were used to examine evidence of horizontal pleiotropy^[20]^.

All statistical analyses were performed using R (version 4.4.1). MR analysis was performed using the “TwoSampleMR” package (version 0.6.8), and MR-PRESSO was conducted using the “MRPRESSO” package. The Benjamini–Hochberg method was used for false discovery rate (FDR) control, and results were considered significant at *P* < 0.05 and *P*_FDR < 0.25.

### LDSC

LDSC regression was used to assess the genetic correlation (*r*_g_) between significant plasma metabolites and breast cancer. The LDSC examined the association between test statistics and linkage disequilibrium to quantify the contribution of inflation from a true polygenic signal or bias^[21]^. The *z*-scores of each variant from Trait 1 were multiplied by the corresponding *z*-scores of each variant from Trait 2. The genetic covariance was estimated by regressing this product on the LD score^[22]^. The genetic covariance normalized by heritability represents the genetic correlation.

### Mapping genetic instruments to genes

To identify significant plasma metabolite-related gene (PMRG) sets, summary Mendelian randomization (SMR) analysis of genetic instruments and genes was performed. The eQTL summary statistics from peripheral blood were obtained from the eQTLGen consortium^[23]^. This study investigated gene expression at the transcriptional level in peripheral blood from 31 684 individuals (primarily of European descent). eQTLs with a *P-*value < 0.05 in tests were used for further analysis.

### Enrichment analysis

After obtaining the PMRGs, Kyoto encyclopedia of genes and genomes (KEGG) and gene ontology (GO) enrichment analyses were performed to elucidate the potential molecular mechanisms underlying plasma metabolites regulating breast cancer risk. The “clusterProfiler” package was used for functional enrichment analysis, and the “ggplot2” package was used to visualize the top 10 most significant enriched pathways and functions.

### Bulk RNA analysis

To further identify the PMRGs upregulated in tumors, the expression profiling by array of GSE42568 was downloaded from the Gene Expression Omnibus (GEO) database (https://www.ncbi.nlm.nih.gov/geo/)^[24]^. The dataset includes 121 samples, comprising 104 breast cancer and 17 normal breast biopsy tissues. The GPL570 [HG-U133_Plus_2] Affymetrix Human Genome U133 Plus 2.0 Array platform was used to generate the matrix data. The “limma” package was used to identify differentially expressed genes (DEGs), with the criteria for DEGs identification set as *P*-adjusted < 0.05 and FC ≥ 2. GSE29431 and GSE57297 were also used as external cohorts to validate the expression of key PMRGs.

### Single-cell RNA (scRNA) analysis

The immune landscape patterns of key PMRGs in breast cancer were investigated using scRNA sequencing data of GSE195861 from the GEO database^[25]^. The “Seurat” package was used to analyze the scRNA data^[26]^. Cells with < 2500 genes expressed and a mitochondrial gene content >10% were considered low-quality data and excluded. The “NormalizeData” function was used to normalize the data with “LogNormalize” and then converted into a Seurat object. After identifying the top highly variable genes, the first 15 principal components were selected for principal component analysis (PCA). Then, the “RunUMAP” function was used for uniform manifold approximation and projection (UMAP), and cell clustering was performed based on UMAP-1 and UMAP-2. The “SingleR” package performs cell annotation using bulk RNA-seq samples from immune cell populations in GSE107011^[27,28]^. The expression patterns of key PMRGs in various immune cells were visualized on UMAP. Finally, GSE148673 and GSE161529 were used as external validation cohorts^[29,30]^. The external cohort was performed on tumor immune single cell hub 2 (TISCH2) (http://tisch.compbio.cn/home/)^[31]^.

### Tissue sample collection

This study analyzed nine breast cancer samples from patients who underwent surgery in the breast surgery department of our hospital between February 2024 and January 2025, including three cases each of HER2+, triple-negative (TNBC), and luminal subtypes. The key clinical characteristics of primary breast cancer are summarized in Supplementary Digital Content Table S3, available at: http://links.lww.com/JS9/F233.

### Immunohistochemistry

Tissue sections were mounted on slides. Deparaffinization and rehydration were performed using xylene and a graded ethanol series (100%, 95%, 85%, 70%). Antigen retrieval was conducted by incubating sections in a state buffer using microwave heating for 8 minutes. Sections were blocked with 5% normal goat serum in phosphate-buffered saline (PBS) for 2 hours at room temperature to reduce nonspecific binding. Primary antibody was applied and incubated overnight at 4°C in a humidified chamber. After washing with PBS, sections were incubated with secondary antibody for 1 hour at room temperature. Signal detection was performed using DAB substrate for 1–3 minutes, followed by counterstaining with hematoxylin for 1 minute. Sections were dehydrated through graded ethanol and cleared in xylene. The antibodies used were as follows: SPP1 (GB11500, Servicebio, China); CD11c (GB115690, Servicebio, China); CD68 (GB115723, Servicebio, China); CD8 (GB154196, Servicebio, China).

### Multi-fluorescence immunohistochemical staining

Tissue sections were heated at 60°C for 30 minutes and deparaffinized in xylene twice for 10 minutes each and graded ethanol series (100%, 95%, 85%, 70%) for 5 minutes. The sections were immersed in citrate buffer (pH 6.0), heated in a microwave oven on high power for 8 minutes, and then cooled to room temperature. The sections were incubated in an appropriate dilution of the different primary antibodies. Sections were incubated by each antibody at room temperature for 1 hour or overnight at 4°C, washed three times in PBS for 3 minutes each, and after blotting the excess liquid, sections were incubated in DAPI in the dark for 5 minutes, washed in PBS four times to remove the excess 4’,6-diamidino-2-phenylindole (DAPI), mounted with an antifluorescence quenching mounting medium, and observed under a fluorescence microscope. The antibodies used were as follows: SPP1 (GB11500, Servicebio, China); CD11c (GB115690, Servicebio, China); CD68 (GB115723, Servicebio, China); CD8 (GB154196, Servicebio, China).

### Cell culture and macrophage differentiation

Human breast cancer MDA-MB-231 cells were maintained in Dulbecco’s modified Eagle medium (DMEM) supplemented with 10% fetal bovine serum (FBS; Gibco, USA) and 1% penicillin/streptomycin (Thermo Fisher Scientific, USA). THP-1 monocytes (ATCC^®^ TIB-202™) were differentiated into macrophages by treatment with 100 ng/mL phorbol 12-myristate 13-acetate (PMA; Sigma-Aldrich, USA) for 48 hours in RPMI-1640 + 10% FBS, followed by 24 hours of rest in PMA-free medium.

### Lentiviral transduction and generation of stable cell lines

SPP1 overexpression (SPP1-OE), SPP1 knockdown (SPP1-KD), and corresponding control (SPP1-Con for OE, SPP1-ShCon for KD) lentiviral vectors were constructed by Shanghai Genechem Co., Ltd. Stable cell lines expressing SPP1-OE, SPP1-KD, and controls were generated by lentiviral transduction according to the manufacturer’s protocol. Cells stably expressing the transgenes were selected using culture medium supplemented with 10 μg/mL puromycin. Selection was initiated 48 hours posttransduction and maintained for 2 weeks. The sequences used for overexpression and knockdown of specific targets are provided in Supplementary Digital Content Table S4, available at: http://links.lww.com/JS9/F233.

### Western blot analysis

Transfected THP-1 cells were seeded in 10-cm plates at a density of 5 × 10^6^ cells per plate and stimulated with PMA. After 48 hours of PMA treatment, the culture medium was aspirated, and the cells were washed twice with ice-cold PBS. Cells were then lysed using SDS lysis buffer (Beyotime, P0013G, China) for the specified duration. The lysates were heated at 100°C for 10 minutes, and protein concentrations were determined using a BCA Assay Kit (Beyotime, P0012, China). Equal amounts of protein (20 μg) were resolved by sodium dodecyl sulfate-polyacrylamide gel electrophoresis (SDS-PAGE) at 120 V for 70 minutes. Subsequently, separated proteins were electrophoretically transferred onto a 0.22-μm polyvinylidene fluoride membrane. The membrane was blocked for 1 hour at room temperature with 5% (w/v) nonfat dry milk dissolved in TBST. Following blocking, the membrane was incubated with primary antibodies diluted in blocking buffer overnight at 4°C. After five washes with TBST, the membrane was incubated with appropriate horseradish peroxidase (HRP)-conjugated secondary antibodies diluted in blocking buffer for 1 hour at room temperature. Protein bands were visualized using enhanced chemiluminescence (ECL) reagents (Thermo Scientific, 34 580, USA). Band intensity quantification was performed using ImageJ software. The antibodies used were as follows: Osteopontin/SPP-1 Rabbit mAb (ABclonal, A19092, China); GAPDH (Proteintech, 10 494-1-AP, China).

### Clone formation assay

A coculture system was established using breast cancer cells and macrophages. Breast cancer cells were trypsinized, counted, and seeded into 6-well plates (500 cells/well). After 24 hours, differentiated macrophages were added at a 5:1 (macrophage:cancer cell) ratio using 0.4-µm pore Transwell inserts to permit soluble factor exchange without direct contact. For contact-dependent experiments, macrophages were directly co-cultured with cancer cells. Following coculture, Transwell inserts were removed. Cancer cells were gently washed with and replenished with complete DMEM (10% FBS). Cells were cocultured for 10 days, then fixed with 4% paraformaldehyde (15 min) and stained with 0.5% crystal violet (Sigma-Aldrich; 30 minutes). Colonies (>50 cells) were manually counted under a light microscope.

### CCK-8 assay

Differentiated macrophages were added at a 5:1 ratio (macrophage: cancer cell). Macrophages were placed in 0.4-µm Transwell inserts (Corning, 3413, USA). Cocultures were maintained in serum-free DMEM/RPMI (1:1) for 24–72 hours at 37°C. After coculture, Transwell inserts were removed. Breast cancer cells were seeded in 96-well plates (3 × 10^3^ cells/well) and allowed to adhere for 24–72 hours, followed by the addition of 100 μL fresh serum-free medium and 10 μL CCK-8 reagent (Beyotime, C0037, China) per well. Plates were incubated at 37°C for 2−4 h. Absorbance was measured at 450 nm using a microplate reader.

### Transwell assay

Breast cancer cells MDA-MB-231 and macrophages THP-1 were maintained in their respective media. THP-1 monocytes were differentiated into macrophages by treatment with 100 ng/mL PMA for 48 hours, followed by 24 hours of rest in PMA-free medium. Then, 1 μg/mL of mitomycin C was added to the breast cancer cell culture dishes 1 hour before the start of the experiment to inhibit cell proliferation. Breast cancer cells and macrophages were harvested using trypsin/EDTA, washed twice with PBS, and resuspended in serum-free medium. Breast cancer cells (3 × 104 cells in 200 μL serum-free medium) were seeded into the upper chamber of a 24-well Transwell insert (8-μm pore size; Corning, USA). Macrophages in the control group and experimental group (1.5 × 10^5^ cells in 500 μL complete medium) were placed in the lower chamber. Plates were incubated at 37°C under 5% CO_2_ for 48 hours. Photos were acquired under a microscope after crystal violet staining.

### Evaluation of candidate drugs

Candidate drugs that downregulate genes were retrieved from the Drug Signatures Database (DSigDB) (https://tanlab.ucdenver.edu/DSigDB)^[32]^. The database currently contains 22 527 gene sets, including 17 389 drugs covering 19 531 genes.

### Phenome-wide association analysis

To further assess the potential of genes as drug targets and their possible side effects, pheWAS analysis was performed on the AstraZeneca PheWAS Portal (https://azphewas.com/)^[33]^. This was performed on 15 500 binary and 1500 continuous phenotypes in the UK Biobank. Multiple corrections were performed and a threshold of 1e-8 was established to account for the potential for false positives.

## Results

### MR analysis

According to the selection criteria, we obtained 34 837 genetic instruments, all of which had an *F*-statistic >10, indicating that our findings were not influenced by weak instrument bias (Supplementary Digital Content Table S5, available at: http://links.lww.com/JS9/F233).

**Figure 1.**
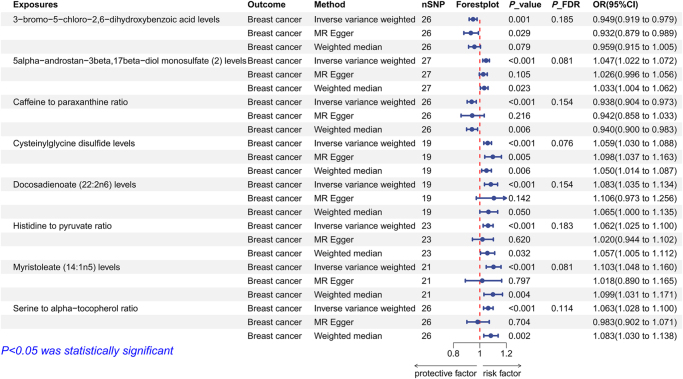
The forest plot of MR estimates for the association between plasma metabolites and breast cancer.

Application of the IVW method resulted in the identification of eight plasma metabolites with a significant causal effect on breast cancer risk (Fig. 1). The analytical results obtained with MR-Egger and weighted median further validated the risk trends observed with IVW. Among them, genetic prediction indicated that six plasma metabolites would increase breast cancer risk, including 5-alpha-androstan-3beta,17beta-diol monosulfate (2) levels, cysteinylglycine disulfide levels, docosadienoate (22:2n6) levels, histidine to pyruvate ratio, myristoleate (14:1n5) levels, and serine to alpha-tocopherol ratio. Genetic prediction indicated that 3-bromo-5-chloro-2,6-dihydroxybenzoic acid levels and caffeine to paraxanthine ratio would reduce breast cancer risk. Supplementary Digital Content Table S6, available at: http://links.lww.com/JS9/F233 exhibit the plasma metabolites with 89 suggestive causal effects. The reverse MR analysis did not identify a bidirectional causal effect between the significant plasma metabolites and breast cancer.

### Sensitivity analysis

**Figure 2.**
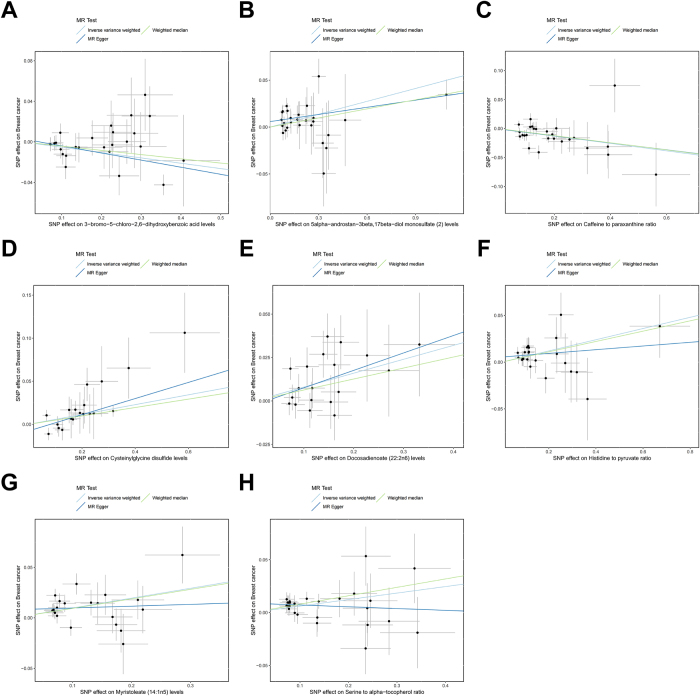
Scatter plots for the causal association between plasma metabolites and breast cancer.

**Figure 3.**
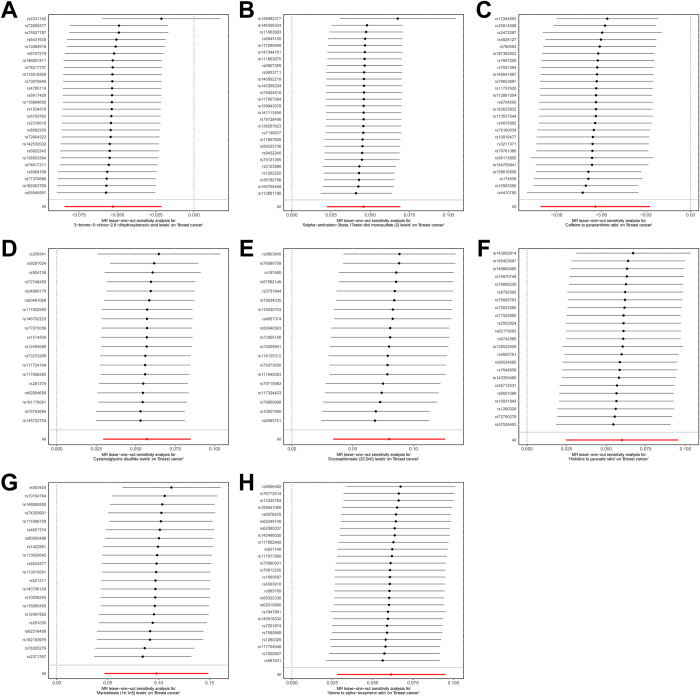
Leave-one-out plots for the causal association between plasma metabolites and breast cancer.

To strengthen the robustness and reliability of the causal effects, we conducted extensive sensitivity analyses. Cochran’s Q test assessed the heterogeneity of the genetic instruments used, which revealed significant heterogeneity for the genetic instrument of the caffeine to paraxanthine ratio. Therefore, we reanalyzed the data using a random-effects IVW while still meeting the significance threshold. A visual inspection of the scatter plot did not reveal any significant outliers (Fig. 2). Leave-one-out analysis further strengthened the results from the scatter plot, with no significant outlier observed (Fig. 3). Subsequently, the Egger intercept test found that the causal effect of 5-alpha-androstan-3-beta,17-beta-diol monosulfate (2) levels might be influenced by horizontal pleiotropy, whereas the MR-PRESSO global test found no evidence of horizontal pleiotropy. This causal effect was not considered robust enough and was excluded from further analysis. The detailed results of the sensitivity analysis can be found in Supplementary Digital Content Table S7, available at: http://links.lww.com/JS9/F233.

### Genetic correlation

LDSC regression was used to assess the genetic correlation between the remaining significant plasma metabolites and breast cancer. Due to the limitation of low heritability, the caffeine to paraxanthine ratio was not used for the above analysis. LDSC regression showed that there was almost no genetic correlation between the remaining six significant plasma metabolites and breast cancer (*P* > 0.05) (Table 1). These findings indicated that the MR estimates were not confounded by common genetic components.

**Table 1:** Genetic correlation between plasma metabolites and breast cancer

Trait1	Trait2	rg	rg_se	*P*_value
3-Bromo-5-chloro-2,6-dihydroxybenzoic acid levels	Breast cancer	−0.065	0.118	0.581
Serine to alpha-tocopherol ratio	Breast cancer	−0.107	0.103	0.299
Cysteinylglycine disulfide levels	Breast cancer	0.205	0.119	0.085
Docosadienoate (22:2n6) levels	Breast cancer	0.167	0.130	0.197
Histidine to pyruvate ratio	Breast cancer	−0.088	0.067	0.193
Myristoleate (14:1n5) levels	Breast cancer	0.390	0.216	0.071
Caffeine to paraxanthine ratio	Breast cancer	NA	NA	NA

rg, genetic correlation; se, standard error.

### Genes and functions

**Figure 4.**
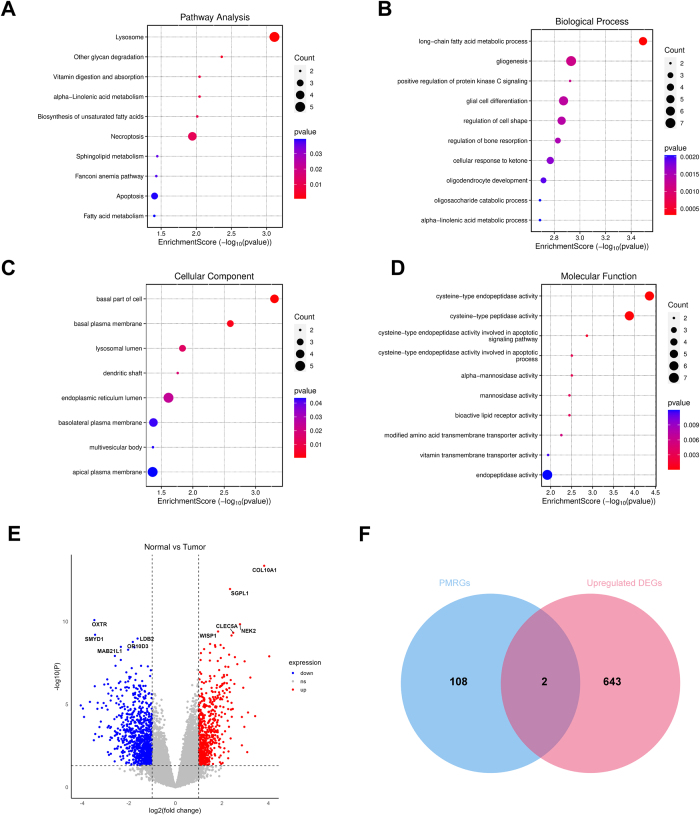
Functional enrichment and identification of upregulated PMRGs. (A) KEGG enrichment analysis. (B–D) Enrichment analysis of biological process, cellular component and molecular function in GO. (E) Differential analysis of the GSE42568 dataset. (F) Venn analysis of the PMRGs set and upregulated genes.

The correspondence between genetic instruments and genes is presented in Supplementary Digital Content Table S8, available at: http://links.lww.com/JS9/F233. KEGG pathway enrichment results showed that the plasma metabolites-related genes (PMRGs) were enriched in pathways such as lysosome, other glycan degradation, and alpha-linolenic acid metabolism (Fig. 4A). GO functional enrichment results showed that PMRGs were associated with functions such as long-chain fatty acid metabolic process and gliogenesis in biological process, basal part of cell and basal plasma membrane in cellular component, and cysteine-type endopeptidase activity and cysteine-type peptidase activity in molecular function (Fig. 4B–D). The detailed results of the KEGG and GO enrichment analyses can be found in Supplementary Digital Content Table S9 and S10, available at: http://links.lww.com/JS9/F233.

### Bulk RNA analysis

To examine the upregulated PMRGs in breast cancer tissues, we used differential analysis on the GSE42568 dataset. Differential analysis showed that the top 10 DEGs between breast cancer and healthy tissues were COL10A1, SGPL1, NEK2, CLEC5A, WISP1, OXTR, SMYD1, LDB2, OR10D3, and MAB21L1 (Fig. 4E). Supplementary Digital Content Fig. S1, available at: http://links.lww.com/JS9/F232, shows the expression of the top 20 DEGs. Venn analysis of PMRGs and upregulated DEGs revealed two key PMRGs that were upregulated in breast cancer, namely SPP1 and ADM2 (Fig. 4F). In two external validation cohorts, the expression level of SPP1 was significantly higher in breast cancer tissue (Supplementary Digital Content Fig. S2, available at: http://links.lww.com/JS9/F232). ADM2 promotes the growth, migration, and invasion of breast cancer cells by inducing Src kinase phosphorylation, thereby triggering c-Myc transcription^[34]^. However, ADM2 was significantly upregulated in breast cancer tissue only in the GSE57297 cohort (Supplementary Digital Content Fig. S3, available at: http://links.lww.com/JS9/F232).

### The scRNA analysis

To observe the immune landscape patterns of key PMRGs, we performed scRNA analysis. Visualization of sequencing depth, gene quantity, and mitochondrial gene content showed that scRNA data were available, as shown in Supplementary Digital Content Fig. S4, available at: http://links.lww.com/JS9/F232. The mitochondrial gene percent was independent of the gene counts, and the sequencing depth was positively correlated with the gene counts, with a coefficient of 0.87 and 0.88 in breast cancer and normal breast tissue (Supplementary Digital Content Fig. S4, available at: http://links.lww.com/JS9/F232).

**Figure 5.**
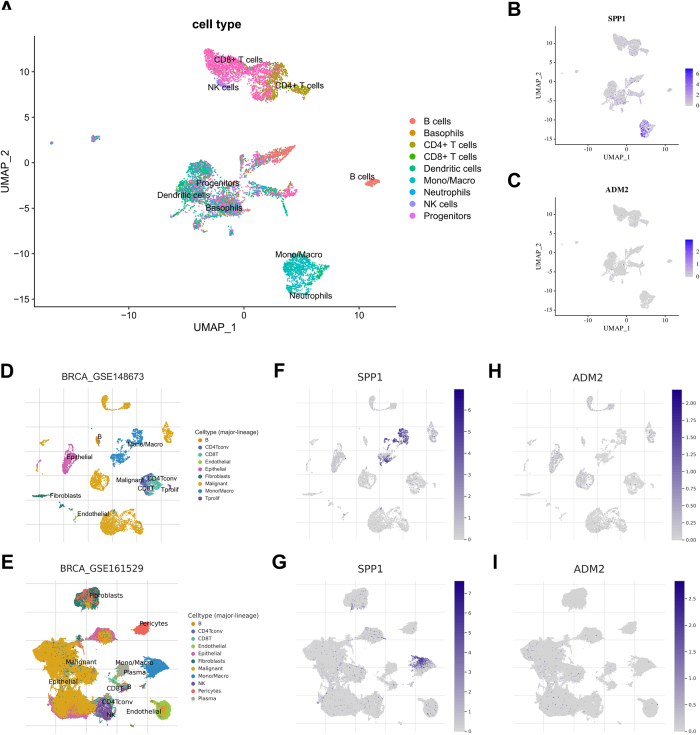
Immune landscape patterns of key PMRGs. (A) Cell annotation of GSE195861. (B and C) Immune landscape patterns of SPP1 and ADM2 in GSE195861. (D and E) Cell annotation of GSE148673 and GSE161529. (F and G) Immune landscape patterns of SPP1 in GSE148673 and GSE161529. (H and I) Immune landscape patterns of ADM2 in GSE148673 and GSE161529.

After preprocessing with quality control, the high-dimensional scRNA data were visualized using UMAP based on the first 15 principal components. The “SingleR” package was used to annotate the data with identifiable cell types. The main cell types included B cells, basophils, CD4 + T cells, CD8 + T cells, dendritic cells, mono/macro, neutrophils, NK cells, and progenitors (Fig. 5A). After projecting the expression levels of key PMRGs onto the UMAP of various immune cells, we found that SPP1 primarily localized to mono/macro, whereas no significant localization of ADM2 was observed (Fig. 5B and C). The main cell types of the two external cohorts were similar to those of the aforementioned cohort (Fig. 5D and E). In the external cohort, SPP1 and ADM2 exhibited similar immune landscape patterns (Fig. 5F–I). Given that the immune landscape pattern of ADM2 was not clearly observed, it was excluded from subsequent analyses. Thus, SPP1 may be a potential target associated with the immune microenvironment.

### SPP1 is mainly distributed in monocytes and macrophages

**Figure 6.**
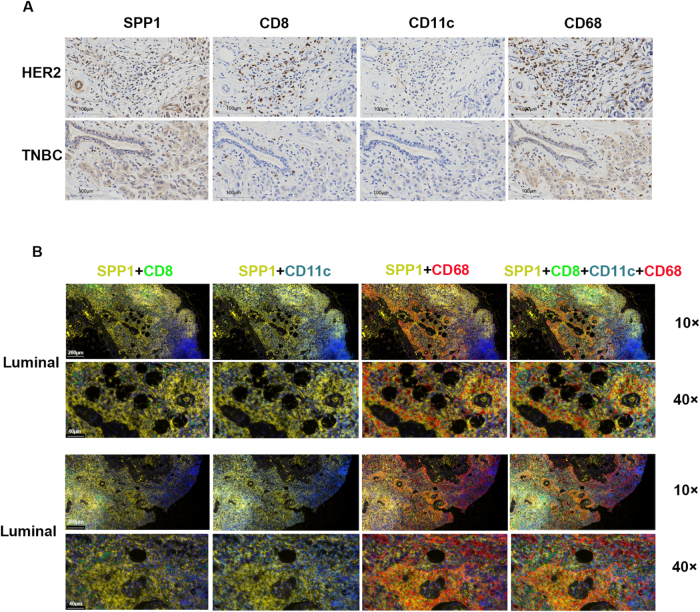
SPP1 is highly expressed in macrophages in various subtypes of breast cancer. (A) SPP1, CD8, CD11c, and CD68-stained images of HER2^+^ and TNBC human tumor tissues; scale bar, 100 µm. (B) The results of multi-fluorescence immunohistochemical staining. Yellow is used for SPP1 staining, green is used for CD8 staining, cyan is used for CD11c staining, and red is used for CD68 staining. The 10× field of view scale is 200 µm, and the 40× field of view scale is 40 µm.

The results of scRNA sequencing indicated that SPP1 was highly expressed in monocytes. Monocytes enter the bloodstream and migrate into tissues, where they differentiate into macrophages^[35]^. Therefore, we verified the results of scRNA sequencing in the tissues. The results of immunohistochemical staining showed that SPP1 was widely and highly expressed in CD68^+^ macrophages, patchily and/or at moderate to low levels in CD11c^+^ DC cells, and was almost undetectable in CD8^+^ T cells. The same trend was observed in tumor tissues of breast cancer patients with HER2^+^ and triple negative breast cancer (TNBC) molecular subtypes (Fig. 6A). The results of multifluorescence immunohistochemical staining showed that SPP1 was mainly distributed in macrophages among interstitial cells, which was also consistent with our single-cell sequencing results (Fig. 6B).

### Macrophage SPP1 promotes the malignant phenotypes of breast cancer cells and is upregulated by myristoleate

**Figure 7.**
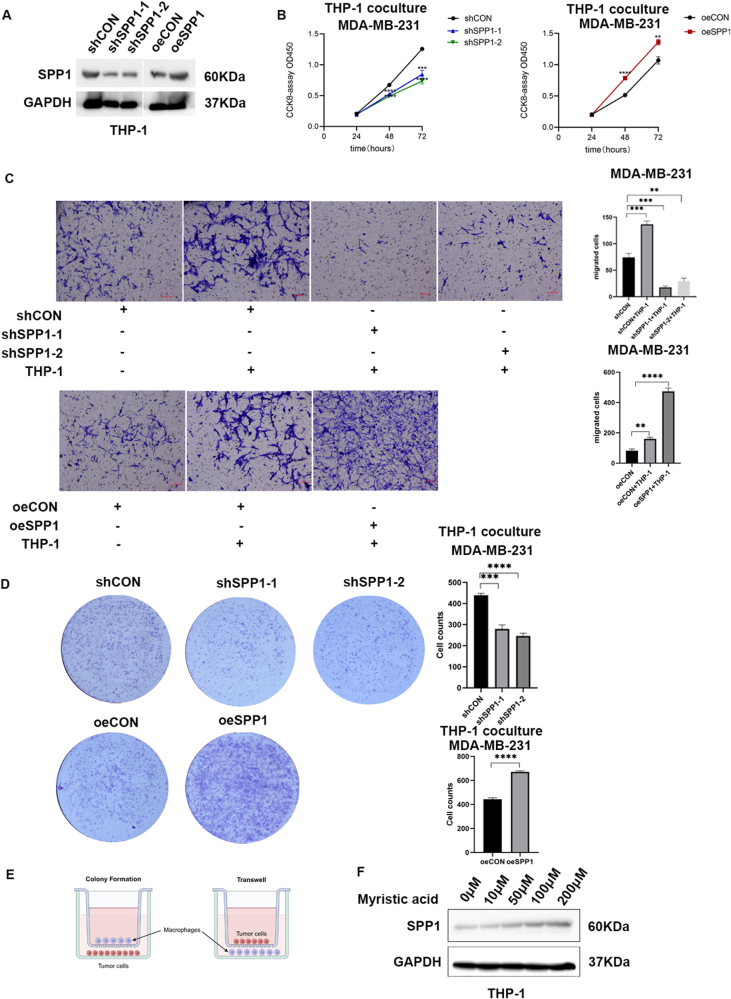
Macrophage SPP1 promotes the malignant phenotypes of breast cancer cells and is upregulated by myristoleate. (A) Establishment of macrophages with stable knockdown and overexpression of SPP1. (B) CCK-8 assay detecting the growth viability of MDA-MB-231 breast cancer cells cocultured with SPP1-knockdown or SPP1-overexpressing macrophages at 24 hours, 48 hours, and 72 hours. (C) Transwell assay detecting the invasion of MDA-MB-231 breast cancer cells after 72 hours of coculture with SPP1-knockdown or SPP1-overexpressing macrophages. (D) Colony formation assay detecting the proliferation of MDA-MB-231 breast cancer cells cocultured with SPP1-knockdown or SPP1-overexpressing macrophages. (E) Schematic diagram of the coculture model. (F) Effect of different concentrations of myristic acid on SPP1 expression after 24 hours of treatment.

To investigate the impact of SPP1 expression on macrophages in breast cancer cells, we differentiated the THP-1 monocytic cell line into macrophages using PMA induction. These macrophages were then subjected to SPP1 knockdown or overexpression and cocultured with MDA-MB-231 cells (Fig. 7A). The effect of macrophages with differential SPP1 expression levels on breast cancer cell proliferation was assessed using CCK-8 and colony formation assays, and their effect on migration was evaluated using Transwell assays. Coculture with SPP1-knockdown macrophages significantly suppressed the proliferation and migration of breast cancer cells, whereas SPP1 coculture with SPP1-overexpressing macrophages promoted the proliferation and migration of breast cancer cells (Fig. 7B–D). Furthermore, to validate the relationship between SPP1 and myristoleate and confirm its effect on regulating SPP1 expression, macrophages were treated with graded concentrations of myristoleate, and SPP1 expression was examined. The results demonstrated that myristoleate upregulated SPP1 expression in macrophages in a dose-dependent manner (Fig. 7E).

### Identification of candidate drugs

SPP1 was analyzed using the DSigDB on Enrichr to identify candidate drugs. The top 10 drugs were ketoconazole, progesterone, ethaverine, atovaquone, dequalinium chloride, papaverine, pyrvinium, bromocriptine, colchicine, and (−)-isoprenaline (Table 2).

**Table 2:** Top 10 candidate drugs predicted using DsigDB

Drug names	*P*-value	Adjusted *P*-value	Genes
Ketoconazole HL60 DOWN	0.002	0.014	SPP1
Progesterone HL60 DOWN	0.002	0.014	SPP1
Ethaverine HL60 DOWN	0.003	0.014	SPP1
Atovaquone HL60 DOWN	0.003	0.014	SPP1
Dequalinium chloride HL60 DOWN	0.003	0.014	SPP1
Papaverine HL60 DOWN	0.003	0.014	SPP1
Pyrvinium HL60 DOWN	0.004	0.014	SPP1
Bromocriptine HL60 DOWN	0.005	0.016	SPP1
Colchicine HL60 DOWN	0.006	0.018	SPP1
(−)-Isoprenaline HL60 DOWN	0.007	0.019	SPP1

DsigDB, drug signatures database.

### PheWAS analysis

**Figure 8.**
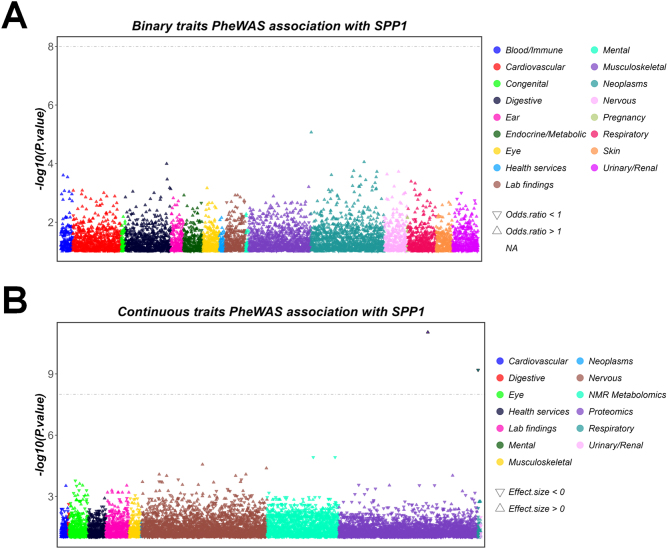
The pheWAS analysis of SPP1. (A) Binary traits. (B) Continuous traits.

To determine whether the downregulation of SPP1 could lead to side effects and capture unrecognized horizontal pleiotropy in sensitivity analysis, we conducted a pheWAS analysis. We identified two continuous traits that were associated with changes in the expression of SPP1. In the European population, the expression level of SPP1 was significantly positively correlated with cardiometabolic traits (Fig. 8A). In the East Asian population, the expression level of SPP1 was significantly negatively correlated with age, hay fever, or allergic rhinitis diagnosed by a doctor (Fig. 8B).

## Discussion

Here, we utilized large-scale GWAS summary statistics to explore the potential causal effects between plasma metabolites and breast cancer, which led to the identification of eight pairs of potential causal effects. Subsequent sensitivity analyses to assess the robustness of these effects showed that only 5-alpha-androstan-3-beta,17-beta-diol monosulfate (2) levels were influenced by horizontal pleiotropy. Reverse MR analysis did not identify any bidirectional causal effects. We mapped the genetic instruments of significant plasma metabolites to genes to provide insight into the potential molecular mechanisms. Finally, the establishment of the PMRGs set provided a unique perspective for the exploration of preliminary candidate targets for the treatment of breast cancer. Overall, this study provided valuable information that will help develop novel preventive and treatment strategies for breast cancer by utilizing various techniques and clinical sample validation.

Currently, metabolomics provides an effective means for exploring the role of endogenous substance metabolism and the impact of exogenous invasive substances in the progression of diseases. The occurrence and progression of breast cancer are characterized by a common metabolic reprogramming process^[36–38]^. Therefore, metabolites and their potential molecular processes remain important in breast cancer risk assessment and prevention. The compound 3-bromo-5-chloro-2,6-dihydroxybenzoic acid is a halogenated hydroxybenzoic acid belonging to the benzoic acid class. Genetic prediction suggests that it is negatively associated with the risk of breast cancer. A previous MR study that used a more lenient genetic instrument threshold observed similar effects^[39]^. The current literature related to 3-bromo-5-chloro-2,6-dihydroxybenzoic acid is limited. A study on diet and plasma metabolic biomarkers indicated that 3-bromo-5-chloro-2,6-dihydroxybenzoic acid is positively associated with the intake of red meat and milk^[40]^. However, red meat consumption is considered one of the factors contributing to an increased risk of breast cancer^[41]^. In addition, high levels of milk consumption are associated with an increased risk of breast cancer^[42]^. Future investigation should focus on metabolomic cohort studies and explore the interactions with target enzymes. Cysteinylglycine disulfide levels are closely related to the cysteinylglycine metabolism pathway. Cysteinylglycine is an oxidant produced during the catabolism of glutathione, which induces oxidative stress and lipid peroxidation^[43]^, and oxidative stress is a risk factor for breast cancer^[44]^. This study also found that two unsaturated fatty acids, docosadienoate (22:2n6) and myristoleate (14:1n5), were associated with an increased risk of breast cancer. The structure of unsaturated fatty acids suggests their potential anti-inflammatory properties^[45]^. However, a study in rats indicated that excessive intake of unsaturated fatty acids could lead to oxidative and inflammatory instability in the body^[46]^. Further experiments are required to elucidate the exact molecular mechanisms involved.

Caffeine to paraxanthine ratio is an important pharmacokinetic indicator used to reflect an individual’s capacity to metabolize caffeine. It primarily assesses the caffeine metabolism rate by comparing the concentrations of caffeine and its main metabolite paraxanthine in the blood. Coffee consumption and caffeine intake may be associated with a reduced risk of breast cancer^[47,48]^. These results are consistent with the present findings. Histidine to pyruvate ratio is an indicator of the status of energy metabolism and amino acid metabolism in the body. The catabolism of histidine and other free aromatic amino acids is one of the preferred pathways supporting tumor growth. Previous metabolomics studies reported a significant decrease in the levels of histidine in breast cancer^[49,50]^. This may be due to the increased demand for histamine in breast cancer^[51]^. Meanwhile, histidine is a precursor of histamine, which plays an important role in tumor growth, migration, and invasion^[52]^. In this study, the histidine to pyruvate ratio was positively correlated with breast cancer risk. Future studies should further elucidate the specific role of this ratio in the occurrence and development of breast cancer. The serine to alpha-tocopherol ratio also affected breast cancer risk in this study. p53 mutant breast cancer cells support rapid growth by promoting the synthesis of serine^[53]^, this effect is also supported through the overexpression of phosphoglycerate dehydrogenase^[54]^. Alpha-tocopherol is an important antioxidant that may potentially reduce the risk of breast cancer^[55]^. A cohort study indicated that the intake of alpha-tocopherol is associated with a reduced risk of breast cancer in postmenopausal women^[56]^. Experimental studies have also shown that alpha-tocopherol increases cell apoptosis and decreases cell proliferation^[57,58]^. Although direct evidence of the role of the serine to alpha-tocopherol ratio in breast cancer is currently lacking, both early studies and the present study suggest that its impact on breast cancer risk is multifaceted.

In the established PMRG set, SPP1 was identified as a preliminary candidate target in breast cancer through various bioinformatics techniques. A previous TCGA cohort study showed that SPP1 is significantly increased in breast cancer and associated with poor prognosis^[59]^. SPP1 is closely associated with tumor-associated macrophages, and SPP1 + macrophages are associated with poor prognosis^[60]^. This may be related to the interaction between secreted SPP1 and CD44 on the surface of tumor cells, which activates the PDE3B pathway^[61]^. Functional studies suggest that the reduction of SPP1 inhibits breast cancer cell proliferation and promotes cell apoptosis^[62,63]^. In addition, SPP1 is involved in the remodeling of the breast cancer microenvironment. SPP1 + macrophages promote extracellular matrix remodeling and matrix fibrosis by producing higher levels of transforming growth factor β, interleukin-1β, and SPP1 under hypoxic conditions^[64]^. Overall, SPP1 is involved in various stages of breast cancer development, supporting its value as a potential candidate target. Nevertheless, further exploration is needed regarding the upstream regulatory factors of SPP1 (including plasma metabolites and other molecular factors). SPP1 and myristoleate (14:1n5) levels are influenced by the same SNP (rs62316458), and their potential causal relationship was further explored. SPP1 expression in macrophages increased with rising myristoleate concentrations, demonstrating a dose-dependent response.

This study had several limitations. First, the significance threshold of the traditional GWAS (*P* < 5e-08) was relaxed, which increased the possibility of false positives. Second, we only included a subset of significant plasma metabolites in the subsequent analysis, which may have led to the exclusion of valuable information. Finally, the GWAS data were derived from individuals of European ancestry, which may limit the generalizability of our results to other populations. To partly address these issues, we used an FDR control in the study to reduce the false positive rate. In the future, genetic instruments for the suggestive plasma metabolites should be mapped to facilitate subsequent analyses. Most importantly, extensive GWAS analyses of plasma metabolites in other ancestries should be conducted to ensure the generalizability of the results.

## Conclusion

This study used MR and LDSC regression to examine the causal effects between plasma metabolites and breast cancer from a genetic perspective. Additionally, eQTLs aided in the construction of the PMRG set. Finally, a series of steps, including bulk RNA, scRNA, pheWAS, and clinical sample validation, identified SPP1 as a preliminary candidate target. The results provided insight for the development of personalized treatment strategies for breast cancer and preliminary candidate targets and drugs for future drug development.

## Data Availability

The datasets presented in this study can be found in online repositories. The names of the repository/repositories and accession number(s) can be found in the article.

## Research registration unique identifying number (UIN)

Name of the registry: not applicable. Unique identifying number or registration ID: not applicable. Hyperlink to your specific registration (must be publicly accessible and will be checked): not applicable.
